# Validation of Ultrasound Measurement of Vastus Lateralis for Appendicular Skeletal Muscle Mass in Chronic Kidney Disease Patients with Hemodialysis

**DOI:** 10.3390/diagnostics14222600

**Published:** 2024-11-20

**Authors:** Peng-Ta Liu, Ta-Sen Wei, Congo Tak-Shing Ching

**Affiliations:** 1 Graduate Institute of Biomedical Engineering, National Chung Hsing University, Taichung 402, Taiwan; d109068001@mail.nchu.edu.tw; 2 Fall Prevention Center, Department of Physical Medicine & Rehabilitation, Changhua Christian Hospital, Changhua 500, Taiwan; 3 Department of Electrical Engineering, National Chi Nan University, Nantou 545, Taiwan; 4 International Doctoral Program in Agriculture, National Chung Hsing University, Taichung 402, Taiwan; 5 Advanced Plant and Food Crop Biotechnology Center, National Chung Hsing University, Taichung 402, Taiwan; 6 Doctor Program in Tissue Engineering and Regenerative Medicine, National Chung Hsing University, Taichung 402, Taiwan; 7 Department of Health and Physical Education, The Education University of Hong Kong, Hong Kong SAR, China

**Keywords:** muscle thickness, muscle mass, echo intensity, sarcopenia, ultrasound

## Abstract

Background: Chronic kidney disease patients undergoing hemodialysis (HD) are at a high risk of developing sarcopenia. This study aimed to validate the performance of ultrasound (US) measurements of the vastus lateralis (VL) for estimating muscle mass and diagnosing sarcopenia in CKD patients with HD. Methods: Forty-six patients were enrolled in this study. Muscle thickness (MT) and echo intensity (EI) of VL, physical performance, and biochemical markers were collected to establish a linear regression model for predicting appendicular skeletal muscle mass (ASM), using dual-energy X-ray absorptiometry (DXA) as the reference standard. The model’s performance was validated, and its diagnostic accuracy for sarcopenia was also evaluated. Results: An ASM prediction model was derived: −20.17 + 1.90 × MT_VL (cm) + 1.58 × male + 0.16 × Height (cm) + 0.09 × Weight (kg) + 0.05 × Age (year), with a standard estimated error of 1.44 kg and adjusted R-squared of 0.84. The model exhibited high correlation and an acceptable limit of agreement, compared to DXA measurement. EI displayed a negative correlation with ASM and MT. Conclusions: The ASM adjusted with BMI demonstrated superior performance in diagnosing sarcopenia compared to the ASM adjusted with height. Ultrasound provides a cost-effective bedside tool for evaluating muscle conditions in HD patients.

## 1. Introduction

Sarcopenia is recognized as a progressive loss of skeletal muscle mass, strength, and reduced functional performance. Chronic kidney disease (CKD) patients are particularly vulnerable to sarcopenia due to metabolic abnormalities, inflammation, malnutrition, and physical inactivity [1,2]. Patients with CKD on hemodialysis (HD) further exacerbate muscle loss by causing repeated fluctuations in fluid and electrolyte balance, inducing catabolic states, and promoting oxidative stress [3]. The prevalence of sarcopenia in patients with CKD on HD ranges from 8% to 39.5%, depending on the consensus used and the characteristics of the study population [4,5,6,7]. Early identification of sarcopenia in patients with CKD on HD is critical for appropriate exercise or nutritional interventions to preserve muscle mass, strength, and function. Ultimately, this proactive strategy can significantly improve their quality of life and reduce morbidity and mortality rates.

Sarcopenia is assessed by evaluating muscle mass, strength, and physical performance. Muscle mass measurements include dual-energy X-ray absorptiometry (DXA), computed tomography (CT), and bioelectrical impedance analysis (BIA) [8]. DXA and CT offer accurate muscle mass measurements, but may be costly or inaccessible. Bioelectrical impedance analysis offers a convenient and non-invasive method for estimating body composition. However, its accuracy may be compromised by hydration status, and the equations used are often unreliable in the CKD population [9].

Ultrasound, a non-invasive imaging modality, offers several advantages, including accessibility, affordability, and non-radiation exposure. It not only enables real-time imaging and assessment of muscle architecture, but it also provides data on muscle quality [10]. Various ultrasound-derived equation models have been reported that are applicable to predict body muscle mass in the healthy population [11,12]. Ultrasound-derived parameters, such as muscle thickness, cross-sectional area, and shear wave velocity, have emerged as promising tools for predicting muscle mass and diagnosing sarcopenia in CKD [6,13,14,15].

Thigh muscle thickness is commonly used as a surrogate measure to predict muscle mass. Studies by Matsuzawa et al. [14] and Wilkinson et al. [16] demonstrated that the cross-sectional area of the rectus femoris predicts muscle mass in HD patients, while Rao et al. [15] found a significant correlation between the muscle thickness of the rectus femoris and appendicular skeletal muscle mass (ASM) in predialysis CKD patients. Furthermore, Battaglia et al. [17] observed a reduction in rectus femoris thickness in HD patients, and Wu et al. [18] highlighted a correlation between high echo intensity of the rectus femoris and low physical performance in HD patients. Unlike the rectus femoris, which comprises only 15% of the quadriceps, the VL constitutes the largest and most powerful muscle in the thigh. The location of VL allows for convenient ultrasound probe placement, even in patients lying in bed during dialysis. However, research on ultrasound-derived measurements of the thickness of the vastus lateralis muscle in patients with CKD on HD remains limited. Additionally, the assessment of muscle quality using echo intensity is rarely discussed. More importantly, these crucial muscle parameters are not available in dual-energy X-ray absorptiometry (DXA) or bioelectrical impedance analysis (BIA). Furthermore, there is currently no established consensus regarding the use of VL for diagnosing sarcopenia.

This study aimed to fill the existing knowledge gaps by developing a predictive model for muscle mass. This model will utilize ultrasound measurements of the vastus lateralis muscle thickness and will be validated against dual-energy x-ray absorptiometry measurements of appendicular muscle mass. Additionally, we will assess the effectiveness of this predictive model in diagnosing sarcopenia in patients with CKD on HD, according to various consensus definitions. Importantly, this research has the potential to offer a non-invasive and cost-effective approach for the early detection and management of sarcopenia in patients with CKD on HD, thereby enhancing their quality of life.

## 2. Materials and Methods

### 2.1. Subjects

All subjects were recruited from the dialysis department of a medical center hospital (Changhua Christian Hospital) in Taiwan. Inclusion criteria comprised individuals aged 50 years and above who had been undergoing hemodialysis for more than 3 months with end-stage renal disease. Exclusion criteria included recent hospitalization within the last 3 months, active cancer treatment, severe heart failure, severe liver cirrhosis, and a history of fractures involving the spine or lower extremities. Informed consent was obtained from all participants prior to enrollment of study. This study received ethical approval (CCH-170208), and its corresponding registration code was recorded on ClinicalTrials.gov under NCT03798418.

### 2.2. Standardization of Assessment Procedures

All assessments were arranged in the morning, on fasting, and before dialysis on the same day. The sequence of assessment was as follows: blood sample collection, DXA, ultrasound, and physical performance. Except for blood tests, all examinations are conducted by designated specialists (a radiologist, a rehabilitation physician, and a physical therapist) to ensure consistency in data collection. Each patient was given at least a 10 min break between examinations.

### 2.3. Dual-Energy X-Ray Absorptiometry (DXA) Measurement

The composition and percentage of body components, including lean body mass and fat mass, were measured using DXA (Hologic Delphi QDR, MA, USA). Participants were positioned supine on the DXA scanning table, with the measurement process taking approximately 30 min. Muscle mass in the four limbs was segmented based on operator-defined cutlines at specific anatomical landmarks. Appendicular skeletal muscle mass (ASM) was then calculated as the sum of the lean mass from both arms and legs.

### 2.4. Muscle Ultrasound Measurements

**Figure 1:**
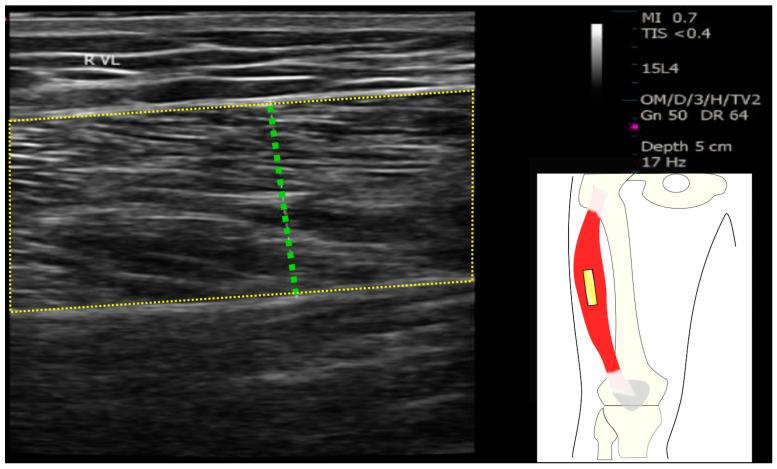
Ultrasound measurement and transducer placement on the vastus lateralis. The muscle thickness of the vastus lateralis was depicted by the green dotted line, while the echo intensity was obtained from the yellow region of interest.

Subjects were positioned supine on the examination bed with their legs extended and rested for 5 min. A prefabricated foot splint was applied to the right ankle to maintain the foot vertically and ensure leg muscle relaxation during the measurement. US measurements were performed and analyzed by a physician with over 10 years of experience to ensure the accuracy of data collection. The ultrasonic image of the right vastus lateralis muscle was acquired at distal 65% between the greater trochanter and the lateral femoral condyle [19] and marked with a color pen (Figure 1).

A high-resolution 2D/B-mode broadband ultrasonic instrument (T3300, BENQ, Taoyuan, Taiwan) with a linear probe (3–17 MHz) was utilized. All US scanning parameters were kept constant for each subject. Gain was set at 50% of the range, the dynamic range was kept at 64 dB, time gain compensation was kept, and the depth was set at 5 cm. The midpoint of transducer was aligned with the predefined mark of the VL and long-axis of transducer was placed longitudinally along the VL. The operator applied a gentle pressure when placing the transducer vertically on the skin. Three images were acquired and stored for offline analysis by using Image J software version 1.51s (National Institutes of Health, Bethesda, MD, USA) to quantify muscle thickness and echo intensity. MT of VL was defined as the distance between the superficial and deep aponeuroses. Echo intensity was calculated by selecting a maximum rectangular region of interest (ROI) within the VL as shown in the US image and obtaining the mean pixel value of this ROI. The average of the three measurements was used for subsequent statistical analysis.

### 2.5. Biochemical Tests

To enhance understanding of the relationship between biochemical tests and muscle quality and quantity, we also collected fasting morning blood samples for the analysis of albumin, cholesterol, creatinine, C-reactive protein, and estimated glomerular filtration rate.

### 2.6. Physical Performance

The grip strength test was performed with a handheld electronic grip dynamometer (Jamar Plus+; Sammons Preston, Rolyon, Bolingbrook, IL, USA). The subjects held the dynamometer with their right hand with the elbow flexed at 90 degrees alongside their body. Maximal grasp force was measured three times, and the average value was recorded. For the Timed Up and Go (TUG) test [20], participants began seated in a standard arm-chair with a straight back and hands resting on their knees. Upon receiving a “start” signal, they were instructed to walk 3 m to a designated line on the floor and return to sit back down in the chair. Time was recorded using a digital stopwatch. The test was performed three times, and the average time was calculated.

### 2.7. Sarcopenia Evaluation

Sarcopenia was diagnosed based on the international consensus from the Asian Working Group for Sarcopenia (AWGS), the European Working Group on Sarcopenia in Older People (EWGSOP2), and the Foundation of the National Institutes of Health (FNIH). Sarcopenia was defined by the presence of low muscle mass and low muscle strength. The specific cutoff values for muscle mass and muscle strength for each consensus are as follows:

AWGS: Low muscle mass is defined as the appendicular skeletal mass index (ASMI, kg/m^2^), calculated as appendicular skeletal mass divided by height squared. Muscle mass was considered low when ASMI was below 7.0 kg/m^2^ for males and 5.4 kg/m^2^ for females. Low muscle strength is indicated by handgrip strength <28 kg for males and <18 kg for females for AWGS [21].

EWGSOP2: Low muscle mass is defined as ASMI <7.0 kg/m^2^ for males and <5.5 kg/m^2^ for females. Low muscle strength is indicated by handgrip strength <27 kg for males and <16 kg for females for EWGSOP2 [22].

FNIH: Low muscle mass is defined as ASM <19.75 kg for males and <15.02 kg for females, or ASM divided by BMI (ASM/BMI) <0.789 kg/m^2^ for males and <0.512 kg/m^2^ for females. Low muscle strength is indicated by handgrip strength <26 kg for males and <16 kg for females [23].

### 2.8. Statistical Analysis

The descriptive statistics for continuous variables were calculated as mean values ± standard deviation, while categorical variables were expressed as numbers and percentages. Pearson’s correlation (*r*) was used to determine the relationships between muscle quantity, muscle quality, biochemical tests, and physical performances.

Python version 3.12 (Python Software Foundation, Wilmington, DE, USA) was utilized to develop and validate a predictive model for muscle mass. A statistical significance threshold of *p* < 0.05 was set for all analyses. The stepwise method was used to develop the ASM prediction formula. The standard error of estimation (SEE) was used to assess the model accuracy. Model performance was validated using the LeaveOneOut cross method.

To evaluate the agreement between two clinical measures of ASM obtained from DXA-measured and US-derived muscle mass, the Bland–Altman analysis was conducted [24]. Lin’s concordance correlation coefficient (CCC) was also calculated to assess the agreement between muscle mass obtained from DXA and those predicted from the US. CCC ranges from –1 to 1, with a perfect strength-of-agreement considered as 1 [25]. The receiver operating characteristic (ROC) curve analysis was used to evaluate the performance of the sarcopenia classification model. The area under the curve (AUC), sensitivity, specificity, accuracy, precision, and F-score were calculated for model comparisons across different sarcopenia consensuses used in this study.

## 3. Results

This study included 46 HD patients, comprising 25 males (54.3%) and 21 females (45.7%), with an overall mean age of 67.3 ± 11.0 years. Males exhibited significantly higher ASM (19.9 ± 2.5 kg and 14.4 ± 2.0 kg, *p* < 0.001) and ASMI (7.2 ± 0.7 kg/m^2^ and 6.2 ± 0.8 kg/m^2^, *p* < 0.001), compared to females. Additionally, the VL muscle thickness (1.8 ± 0.4 cm vs. 1.5 ± 0.4 cm, *p* = 0.017) and grip strength (23.06 ± 6.06 kg vs. 14.55 ± 4.96 kg, *p* < 0.001) were also significantly greater in males. No significant differences were observed between sex in EI, gait speed, and TUG. The overall prevalence of sarcopenia ranged from 23.9% (FNIH/BMI) to 52.1% (FNIH), with a higher prevalence observed in males. All patients’ characteristics are presented in Table 1.

### 3.1. Predictive Model of Muscle Mass

The linear regression model for estimation of appendicular skeletal muscle is shown in Table 2. The ASM predictive formula was as follows:

ASM (kg) = −20.17 + 1.90 × Vastus lateralis muscle thickness (cm) + 1.58 × Sex + 0.16 × Height (cm)

+ 0.09 × Weight (kg) + 0.05 × Age (year)

where sex is 0 if female or 1 if male, and the adjusted R^2^ was 0.84 with SEE of 1.44 kg. The mean SEE from LOOCV for this model was 1.54 kg.

### 3.2. Validation of US-Derived Muscle Mass

**Figure 2:**
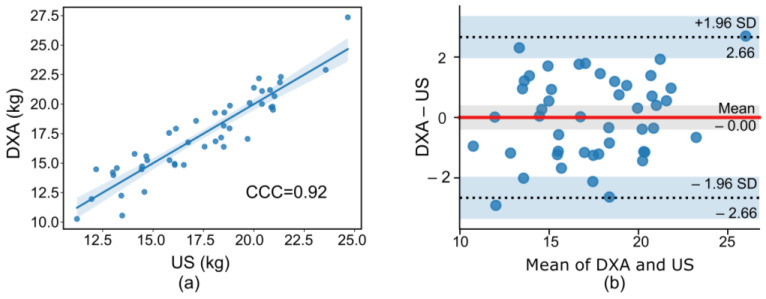
(**a**) Correlation between muscle mass obtained by DXA-measured and US-derived methods. (**b**) Bland–Altman plot comparing muscle mass measurements obtained from DXA and US at the vastus lateralis. The red line represents mean difference. CCC: concordance correlation coefficient, DXA: dual-energy X-ray absorptiometry, US: ultrasound.

The concordance correlation coefficient between MT_VL and DXA-measured muscle mass was highly correlated (r^2^ = 0.92, *p* < 0.001), as shown in Figure 2a. The Bland–Altman plot agreement analysis revealed an acceptable limit of agreement (LOA) of 2.66 kg, with no observed bias (mean difference between DXA and US methods = 0), as shown in Figure 2b.

### 3.3. Accuracy of Diagnosing Sarcopenia

**Figure 3:**
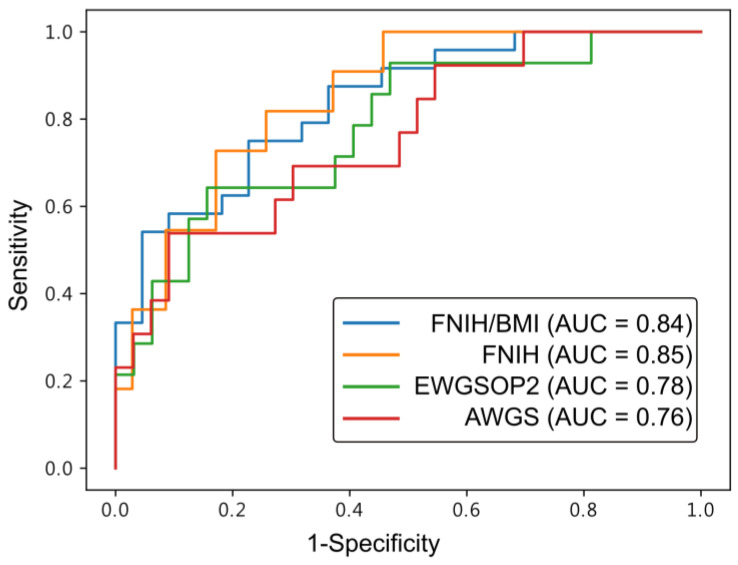
Comparison of receiver operating characteristic (ROC) with area under curve (AUC) among four sarcopenia consensuses.

The performance of the predictive model for diagnosing sarcopenia in HD patients varied among different consensuses, as shown in Table 3. Figure 3 illustrates the diagnostic accuracy of our predictive model across the four consensuses of sarcopenia. The FNIH/BMI and FNIH consensus demonstrated excellent discrimination, with AUC values of 0.84 and 0.85, respectively. Meanwhile, the EWGSOP2 and AWGS consensus showed acceptable discrimination levels, with AUC values of 0.78 and 0.76, respectively. In particular, FNIH/BMI exhibited the highest sensitivity but slightly lower specificity, in contrast to other consensuses that displayed lower sensitivity and higher specificity. FNIH/BMI also achieved the highest precision and the F-score. Overall, the FNIH/BMI consensus provided superior performance in predicting sarcopenia in CKD patients compared to the other consensuses.

**Figure 4:**
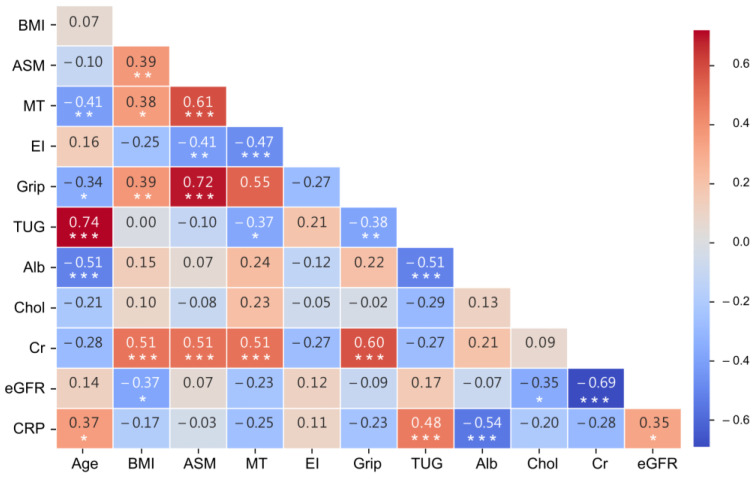
The correlation heatmap between demographic data, biochemical tests, ultrasound muscle parameters, and physical performances. Alb: albumin, ASM: appendicular skeletal muscle mass by DXA, BMI: body mass index, Chol: cholesterol, Cr: creatinine, CPR: C-reactive protein, eGFR: estimated glomerular filtration rate, MT: muscle thickness, EI: echogenicity, TUG: timed up and go. *: *p* < 0.05, **: *p* < 0.01, ***: *p* < 0.001.

Figure 4 illustrates the relationships among DXA-measured AMS and US muscle parameters, biochemical tests, and physical performances. The results revealed that ASM was significantly positively correlated with MT (r = 0.61), EI (r = −0.41), and grip strength (r = 0.72). MT exhibited significant negative correlations with EI (r = −0.47) and TUG (r = −0.37). Creatinine levels showed a moderately positive correlation with MT, ASM, and grip strength (all *p* values < 0.001). Albumin was negatively correlated with TUG (r = −0.37).

## 4. Discussion

This study developed a US-derived appendicular lean mass predictive equation utilizing the vastus lateralis muscle thickness and validated by DXA measurement in patients with CKD on hemodialysis. The results demonstrated a high correlation between US-derived muscle mass and DXA-measured muscle mass, incorporating factors such as sex, age, height, weight, and muscle thickness of VL. The predictive equation exhibited high precision in predicting ASM, with an LOA of 2.6 kg and an SEE of 1.44 kg. The mean SEE of LOOCV was 1.54 kg, and the proposed model explained 84% of the variability of ASM. The precision of the diagnostic model for sarcopenia ranged from excellent to acceptable when using four commonly seen consensus criteria. Echo intensity of the vastus lateralis muscle was found to be correlated with muscle mass and muscle thickness. The FNIH/BMI index was identified as the most suitable indicator for the diagnosis of sarcopenia. The findings supported that the US measurement of VL thickness is a valid predictor for estimating muscle mass and diagnosing sarcopenia in hemodialysis patients.

Previous studies have demonstrated that ultrasound measurements of vastus lateralis muscle thickness serve as a reliable predictor of muscle mass in patients with patellofemoral pain [26], stage 5 chronic kidney disease [27], and osteoarthritis [28]. Therefore, it provides a solid foundation for our single-site muscle mass predictive model. For example, Abe’s study on elderly individuals used six sites, reporting a SEE of 1.43 and an adjusted R-squared value of 0.95 [29]. The study by Paris et al. which used four sites, reported an adjusted R-squared value of 0.72, but with a higher SEE of 2.88 kg [30]. Similarly, Barbosa Silva et al. used a combination of two body lengths (arm and height) and two muscle thickness (arm and thigh) measurements, yielding an adjusted R-squared value of 0.89, and a root-mean-square deviation (RMSE) of 1.3 kg [31]. Our predictive model demonstrates competitive accuracy compared to these past studies, further supporting the potential of using single-site vastus lateralis muscle thickness for predicting muscle mass.

In previous multiple-site prediction models, muscle thickness of the hands and thighs have been commonly utilized alongside demographic variables such as age, sex, and height. In particular, body height or limb length demonstrated significant contributions to the equations to adjust the body size, yielding a lower SEE [29,30,31]. While the inclusion of multiple measurement sites can enhance the explanatory power of data variability and reduce error values, it is crucial to maintain clinical practicality, favoring simplicity [11]. In contrast to the multiple sites approach, Abe et al. proposed a single forearm muscle thickness method to predict muscle mass for older adult populations, yielding SEE values ranging from 1.95 to 2.26 and an adjusted R-squared value between 0.88 and 0.91 [29]. Although assessing forearm muscle thickness may be convenient, it is challenging for HD patients due to the presence of fistulas in the forearm. Additionally, research shows that sarcopenia primarily impacts the lower limbs, especially the proximal regions like the anterior thigh. This evidence supports the use of VL muscle thickness measurement, highlighting its advantages for evaluating muscle condition in HD patients [29,32,33].

Muscle composition in HD patients is influenced by several factors, including abnormal electrolyte and protein metabolism, hydration status, inflammation, fibrosis, and metabolic and hormonal imbalances [5,34,35]. These alterations can lead to changes in muscle quality, indirectly contributing to muscle loss and dysfunction, which are observable through variations in muscle echo intensity [36,37,38]. Our study revealed that echo intensity showed a negative correlation between ASM and MT, suggesting that a decline in muscle quantity is associated with a deterioration in muscle quality. These findings align with previous research [2,18,39]. In particular, changes in muscle quality may occur before or concurrently with muscle mass loss in HD patients, emphasizing the importance of assessing muscle echo intensity as an early indicator of muscle dysfunction. A recent systematic review reported that shear wave elastography offers greater sensitivity for diagnosing sarcopenia in the CKD population [40], which highlights the advantages of ultrasound imaging in evaluating muscle quality—capabilities not possible with techniques like DXA or BIA. The observed associations between albumin and creatinine levels with muscle quantity and physical performance emphasize the need for regular monitoring of muscle health in HD patients. By integrating ultrasound assessments with routine evaluation of nutritional status, healthcare providers can promptly detect changes in muscle condition. This enables the implementation of targeted interventions, such as nutritional supplementation, exercise programs, and medication adjustments, to prevent further muscle loss and dysfunction in HD patients [41].

Past studies have reported significant variation in the prevalence rates of sarcopenia across different diagnostic consensuses. Our study results reveal that the FNIH without adjustment for BMI showed the highest prevalence rate of sarcopenia (52%), compared to other consensus (ranging from 24% to 30%). This discrepancy can be attributed to the lack of normalization by body size (height or BMI), leading to a potential overestimation of sarcopenia prevalence. Therefore, the FNIH criteria without BMI adjustment may not be recommended for diagnosing sarcopenia in hemodialysis patients [9]. Our study confirmed previous findings of reduced prevalence rates of sarcopenia using the FNIH/BMI criteria compared to EWGSOP2 and AWGS [16,35,42]. Additionally, we observed a notable sex disparity, with females exhibiting notably lower prevalence rates under the FNIH/BMI criteria. This may be attributed to the general higher fat percentage of females, as noted in Yoowannakul’s study on Asian women undergoing hemodialysis [43]. Leinig et al. reported that BMI is an indicator of body fat mass and might mask the loss of muscle mass in patients with CKD [44]. Consequently, the FNIH/BMI criteria may underestimate the prevalence of sarcopenia in females in this study. Conversely, Guida et al. indicated EWGSOP2 could be suitable criteria for CKD, although it may also underestimate the prevalence, particularly failing to recognize sarcopenia in individuals with concurrent obesity [3]. Regarding diagnostic accuracy, FNIH/BMI demonstrated a notable sensitivity = 0.79 and acceptable specificity = 0.68 compared to the EWGSOP and AWGSOP consensus in Table 3. The FNIH/BMI model exhibited an AUC of 0.84, comparable to Wilkinson’s finding of accuracy = 0.7 for the FNIH/BMI model based on the cross-sectional area of the rectus femoris in CKD [16]. Recent investigations by Rao on rectus femoris muscle thickness in predialysis CKD patients reported accuracies ranging from 0.628 to 0.748 using the AWGS consensus [15]. The prevalence of sarcopenia varies across subjects based on factors such as age, stage of CKD, nutritional status, and fat percentage, making it challenging to accurately diagnose sarcopenia. These discrepancies reflect the complexity of the condition and emphasize the need for consensus in diagnostic criteria for the diagnosis of sarcopenia.

Considering that slow walking speed and low grip strength are commonly seen in CKD, Dam et al. also highlighted the utility of FNIH/BMI serving as a reliable discriminator for low lean body mass [42]. These results suggest that the appendicular skeletal muscle mass predicted from vastus lateralis (VL) muscle thickness adjusted by BMI (FNIH/BMI) is a valid and potentially effective diagnostic approach for sarcopenia in HD patients, showing promising results and indicating it may be more effective in correctly identifying cases of sarcopenia.

Our findings highlight the critical importance of establishing a consensus of criteria and cutoff values specific to conditions like CKD to enhance the accuracy of sarcopenia screening in clinical practice. It is worth noting that consensus on ultrasound scanning protocols, including measurement sites, locations, and scan timing, remains elusive. This lack of standardization hinders the establishment of ultrasound as a gold standard tool for the diagnosis of sarcopenia. Furthermore, the inconsistency in predicted variables such as appendicular lean mass (aLM), skeletal muscle mass (SMM), and fat-free mass (FFM) complicates data comparisons between studies. Despite these challenges, ultrasound remains a convenient tool for rapid screening and continuous monitoring and informed diagnostic decision making.

This study has several limitations that should be acknowledged. First, the small sample size employed in our research could potentially limit the generalizability of our findings. The small number of participants could not fully capture the heterogeneity present in the CKD population. The absence of a control group further complicates the interpretation of our results. Additionally, our study focused exclusively on subjects with end-stage renal disease, which restricts our understanding of muscle mass changes across different stages of CKD, including predialysis subjects. Future studies with larger, more diverse cohorts are essential to confirm the generalizability of our results and to capture the heterogeneity of the CKD population, including factors such the duration of CKD and dialysis, comorbidities, nutritional status, and lifestyle. A comprehensive understanding of these variations is crucial for early detection and preventive interventions. Despite these limitations, our study offers valuable insights into the capabilities of ultrasound-derived measurements of the muscle thickness of vastus lateralis to estimate muscle mass in HD patients. Moreover, our results provide a foundation for future research aimed at establishing consensus on diagnostic criteria and scanning protocols, thereby improving the assessment of muscle quantity and quality in this population.

## 5. Conclusions

This study developed a robust predictive equation for ultrasound-derived appendicular lean mass using the vastus lateralis muscle thickness, validated in CKD patients on hemodialysis. The results demonstrated a significant correlation with DXA-measured muscle mass, providing high precision in predicting appendicular skeletal mass. The FNIH/BMI criteria also showed acceptable performance in diagnosing sarcopenia. The findings emphasize the need for consensus on ultrasound scanning protocols and diagnostic criteria in future sarcopenia research.

## Figures and Tables

**Table 1:** Demographic data, biochemical tests, ultrasound measurements, and physical performance.

Variables	Overall (*N* = 46)	Female (*n* = 21)	Male (*n* = 25)	*p* Value
Age (years)	67.3 (11.0)	67.5 (10.4)	67.0 (11.7)	0.883
Height (cm)	160.3 (9.1)	152.7 (5.6)	166.6 (6.0)	<0.001
Weight (kg)	61.6 (11.9)	54.2 (10.2)	67.9 (9.5)	<0.001
BMI (kg/m^2^)	23.9 (3.7)	23.2 (4.1)	24.5 (3.2)	0.265
DXA measurement				
Arms (g)	4691.3 (1187.1)	3709.5 (650.3)	5516.1 (859.0)	<0.001
Legs (g)	12,682.8 (2404.2)	10,713.2 (1542.9)	14,337.2 (1615.2)	<0.001
Total fat (g)	16,633.1 (7670.3)	15,757.5 (8748.0)	17,368.6 (6730.9)	0.495
Body fat (%)	26.1 (9.7)	27.7 (12.0)	24.9 (7.2)	0.358
ASM (kg)	17.4 (3.5)	14.4 (2.0)	19.9 (2.5)	<0.001
ASMI (kg/m^2^)	6.7 (0.9)	6.2 (0.8)	7.2 (0.7)	<0.001
Biochemical tests				
Albumin (g/dL)	3.5 (0.3)	3.4 (0.4)	3.5 (0.3)	0.48
Cholesterol (mg/dL)	158.9 (36.8)	174.5 (34.1)	145.8 (34.4)	0.007
Creatinine (mg/dL)	9.6 (1.7)	8.9 (1.6)	10.3 (1.4)	0.003
eGFR (mL/min/1.73 m^2^)	4.9 (0.9)	4.6 (0.9)	5.2 (0.9)	0.029
CRP (mg/dL)	0.4 (0.4)	0.4 (0.4)	0.4 (0.4)	0.704
Ultrasound measurement				
Muscle thickness (cm)	1.6 (0.4)	1.5 (0.4)	1.8 (0.4)	0.017
Echo intensity	91.9 (14.1)	94.8 (13.3)	89.5 (14.5)	0.204
Physical performance				
Grip strength (kg)	18.1 (6.3)	13.8 (4.6)	21.8 (5.2)	<0.001
TUG (s)	9.6 (2.8)	9.6 (2.7)	9.6 (3.0)	0.974
Gait speed (m/s)	1.0 (0.3)	0.9 (0.3)	1.0 (0.3)	0.242
Sarcopenia prevalence				
FNIH/BMI	11 (23.9%)	2 (9.5%)	9 (36.0%)	0.052
FNIH	24 (52.1%)	13 (61.9%)	11 (44.0%)	
EWGSOP2	14 (30.4%)	6 (28.6%)	8 (32.0%)	
AWGS	13 (28.3%)	4 (19.0%)	9 (36.0%)	

ASM: appendicular skeletal muscle, ASMI: appendicular skeletal muscle index, BMI: body mass index, eGFR: estimated glomerular filtration rate, CPR: C-reactive protein, FNIH: Foundation for the National Institutes of Health, EWGSOP2: revised European Working Group on Sarcopenia in Older People, AWGS: Asia Working Group for Sarcopenia., TUG: timed up and go. The data were presented as mean (standard deviation) for continuous variables, and as number (percentage) for categorical variables.

**Table 2:** Linear regression model for the estimation of appendicular skeletal muscle.

Predictors	Coefficient	OR (95% CI)	SEE	*p* Values
Intercept	−20.17	0 (0~0.003)	7.14	0.007
MT_VL (cm)	1.90	6.71 (1.62~27.75)	0.70	0.010
Sex (female = 0, male = 1)	1.58	4.83 (1.12~20.85)	0.72	0.035
Height (cm)	0.16	1.17 (1.07~1.28)	0.04	0.001
Weight (kg)	0.09	1.09 (1.03~1.15)	0.03	0.002
Age (year)	0.05	1.05 (1.00~1.10)	0.02	0.045

MT_VL: muscle thickness of vastus lateralis, SEE: standard error of estimation, OR: odd’s ratio, CI: confidence interval.

**Table 3:** The performance of the US-derived predictive model for diagnosing sarcopenia across four consensuses.

Consensus	Sensitivity	Specificity	Accuracy	Precision	F-Score	AUC
FNIH/BMI	0.79	0.68	0.74	0.73	0.76	0.84
FNIH	0.18	0.97	0.78	0.67	0.29	0.85
EWGSOP2	0.43	0.94	0.78	0.75	0.55	0.78
AWGS	0.23	0.97	0.76	0.75	0.35	0.76

FNIH: Foundation for the National Institutes of Health, EWGSOP2: revised European Working Group on Sarcopenia in Older People, AWGS: Asia Working Group for Sarcopenia, BMI: body mass index, AUC: area under curve.

## Data Availability

The data that support the findings of this study are available from the corresponding author, C.T.-S.C., upon reasonable request.
